# Design of the First in Human Gene Therapy Trial of TLT-101 for Chronic Heart Failure (FIGHT-HF)

**DOI:** 10.1016/j.jacbts.2025.01.019

**Published:** 2025-02-11

**Authors:** Pia Balmaceda, Tara C. Hitzeman, Kikuyo E. Shaw, Lynn M. Kolhepp, Matthew Killeen, James C. Fang, Javed Butler, Adrian F. Hernandez, TingTing Hong, Robin M. Shaw

**Affiliations:** a Nora Eccles Harrison Cardiovascular Research and Training Institute, University of Utah, Salt Lake City, Utah, USA; b London School of Hygiene and Tropical Medicine, University of London, London, England; c Department of Pharmacology and Toxicology, University of Utah College of Pharmacy, Salt Lake City, Utah, USA; d Tikkunlev Therapeutics, Tampa, Florida, USA; e Division of Cardiovascular Medicine, Department of Internal Medicine, University of Utah School of Medicine, Salt Lake City, Utah, USA; f Baylor Scott and White Research Institute, Dallas, Texas, USA; g Department of Medicine, University of Mississippi, Jackson, Mississippi, USA; h Duke Clinical Research Institute, Durham, North Carolina, USA

Heart failure (HF) is a major contributor to global morbidity and mortality, affecting millions worldwide and imposing a substantial economic burden on health care systems. Despite advancements in pharmacologic therapies, device development, and cardiac rehabilitation programs, HF prognosis remains suboptimal. Patients with HF experience progressive functional decline, leading to increased disability and recurrent hospitalizations for disease exacerbations. Conventional therapies target neurohormonal pathways and systemic stressors, and advanced interventions such as ventricular assist devices and implantable cardioverter-defibrillators act as bridges to transplantation, but all fail to directly target the core pathophysiological mechanism of HF: cardiac muscle dysfunction.

**Figure 1:**
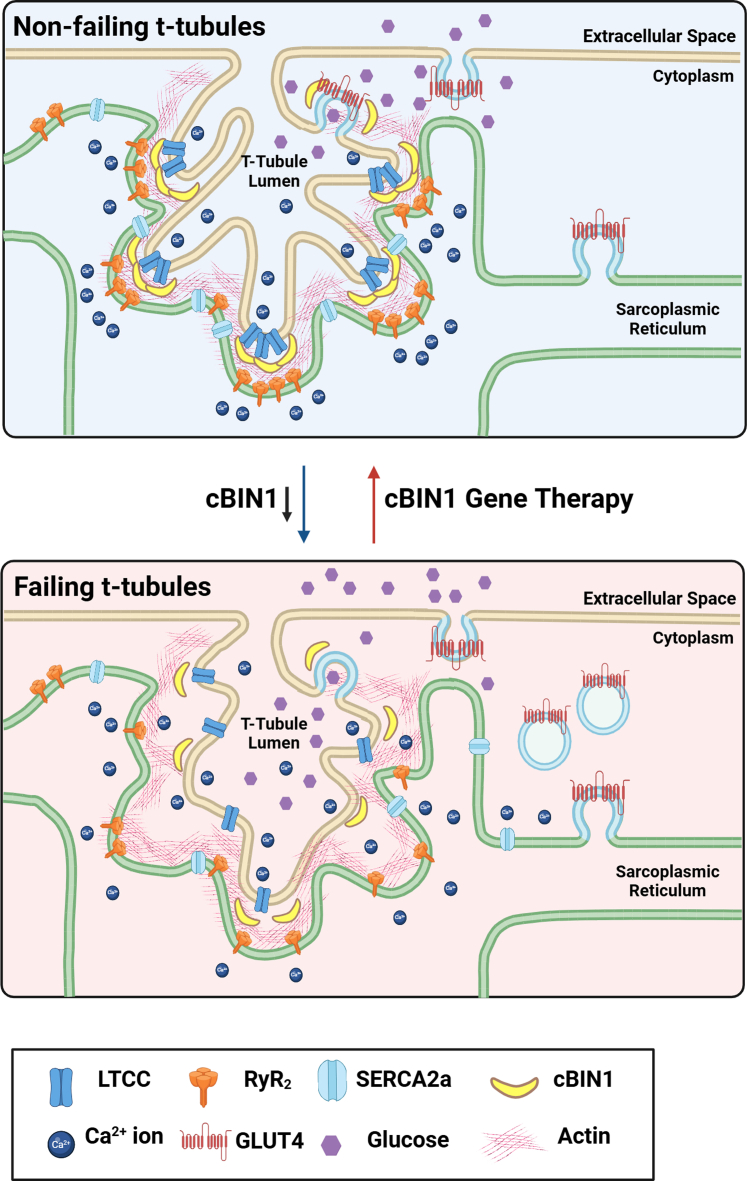
cBIN1 is Responsible for T-Tubule Microarchitecture and Cardiac Function In healthy cardiomyocytes, cardiac bridging integrator 1 (cBIN1) generated microdomains of T-tubule membrane regulate intracellular calcium handling by clustering of L-type calcium channels (LTCCs), ryanodine receptors (RyRs), and SERCA2a, as well as Glut4 glucose transporters. cBIN1 is down-regulated and the clustering is disrupted in failing cardiomyocytes. Gene therapy with exogenous cBIN1 restores cBIN1 microdomains and fidelity of the cardiac calcium transient, resulting in improve inotropy and lusitropy. Created in BioRender.

Cardiac bridging integrator 1 (cBIN1) is responsible for generating T-tubule (TT) membrane microdomains that localize the proteins necessary for a normal and well-organized cardiac calcium transient (Figure 1). cBIN1 levels are transcriptionally down-regulated across all HF subtypes caused by hemodynamic stress.^1^ Reduced cBIN1 expression leads to TT remodeling, characterized by microdomain loss and a disorganized, less-dense TT membrane structure. The loss of TT microdomains results in disorganization of the calcium handling apparatus, impaired calcium handling, weakened and prolonged calcium transients, reduced myocyte contractility, and increased susceptibility to arrhythmias.^2^

TLT-101 is a gene therapy employing a recombinant adeno-associated virus (AAV) vector to deliver the human BIN1 cardiac isoform gene to cardiomyocytes. Preclinical studies have demonstrated the therapeutic efficacy of restoring cBIN1 expression. In murine models of HF induced by chronic isoproterenol infusion and transverse aortic constriction, cBIN1 gene therapy normalized cBIN1 protein levels, repaired TT microarchitecture, and improved cardiac function and survival. These benefits were mediated by cBIN1’s role in enhancing inotropy and lusitropy through the LTCC-RyR dyad formation as well as recovery and, even more importantly, normalization of intracellular sarcoplasmic reticulum ATPase (SERCA2A) localization.^3^

Large animal studies further validated the therapeutic potential of AAV-cBIN1. In a validated porcine model of nonischemic dilated cardiomyopathy and chronic HF induced by tachycardia, a single intravenous dose of AAV-cBIN1 (6 × 10^11^ vg/kg) was administered via the ear vein. Treated animals induced rapid and durable recovery of cardiac function, including improvements in left ventricular ejection fraction, reverse remodeling of chamber size and wall thickness, as well as enhanced survival. The safety profile was favorable, with no significant changes in blood chemistry, including liver function tests. Histopathology revealed no abnormalities aside from mild pulmonary and hepatic congestion linked to advanced disease progression. Six months postadministration, treated animals expressed exogenous cBIN1 protein at this low systemic dose.^4^

The cBIN1 score, an inverse dimensionless index derived from plasma concentrations, in clinical studies of HF patients correlates strongly with HF severity and outperforms N-terminal pro–B-type natriuretic peptide as a prognostic biomarker, accurately predicting future cardiovascular hospitalizations.^5^

The therapeutic potential of TLT-101 will now be evaluated in a nonrandomized, single-arm, open-label dose-escalation trial using a classic 3 + 3 design. Patients with symptomatic nonischemic dilated cardiomyopathy, left ventricular ejection fraction <35%, and NYHA functional class III or American College of Cardiology/American Heart Association (ACC/AHA) Stage C will be enrolled. TLT-101 will be delivered systemically as a single slow infusion through a peripheral vein catheter. Dose escalation and expansion decisions will be guided by an independent Data Safety Monitoring Board. Subjects will undergo a 52-week observation period, with extended safety monitoring continuing for an additional 4 years.

The primary objectives are to assess the safety and tolerability of a single systemic dose of TLT-101 and to determine the recommended phase 2 dose based on adverse events graded according to the Common Terminology Criteria for Adverse Events version 6.0. Exploratory objectives include evaluations of biological activity, biomarkers, functional status, and quality of life. Cardiac response will be assessed with serial echocardiography and periodic cardiac magnetic resonance imaging to examine size, ventricular volume, and systolic and diastolic function. Valve function and atrial volumes will also be measured. Functional status will be evaluated using NYHA functional classification and cardiopulmonary exercise testing. Patient-reported outcomes will be assessed using the World Health Organization Disability Assessment Schedule 2.0 and the Kansas City Cardiomyopathy Questionnaire. Biomarkers, including N-terminal pro–B-type natriuretic peptide and cBIN1 score, will measure myocardial stress and remodeling.

The TLT-101 trial represents an effort to validate the bench-to-bedside translation of an innovative approach targeting a central upstream architectural organizer of the cardiac calcium transient and systolic and diastolic function, marking a promising advance in gene therapy for HF by utilizing a clinically validated cardiotropic viral vector. There is a favorable safety profile consistent with low-dose AAV. Supported by robust basic biology and preclinical research, this trial aims to restore t-tubule architecture to recover left ventricular function and reverse remodel failing hearts, offering a novel therapeutic option for patients with HF and providing clinicians with an additional treatment strategy.
